# Probabilistic PCA of censored data: accounting for uncertainties in the visualization of high-throughput single-cell qPCR data

**DOI:** 10.1093/bioinformatics/btu134

**Published:** 2014-03-10

**Authors:** Florian Buettner, Victoria Moignard, Berthold Göttgens, Fabian J. Theis

**Affiliations:** ^1^Institute of Computational Biology, Helmholtz-Zentrum München, 85764 Neuherberg, Germany, ^2^Department of Haematology, University of Cambridge, Cambridge Institute for Medical Research and Wellcome Trust & MRC Cambridge Stem Cell Institute, Cambridge CB2 0XY, UK and ^3^Department of Mathematics, TU München, 85748 Garching, Germany

## Abstract

**Motivation:** High-throughput single-cell quantitative real-time polymerase chain reaction (qPCR) is a promising technique allowing for new insights in complex cellular processes. However, the PCR reaction can be detected only up to a certain detection limit, whereas failed reactions could be due to low or absent expression, and the true expression level is unknown. Because this censoring can occur for high proportions of the data, it is one of the main challenges when dealing with single-cell qPCR data. Principal component analysis (PCA) is an important tool for visualizing the structure of high-dimensional data as well as for identifying subpopulations of cells. However, to date it is not clear how to perform a PCA of censored data. We present a probabilistic approach that accounts for the censoring and evaluate it for two typical datasets containing single-cell qPCR data.

**Results:** We use the Gaussian process latent variable model framework to account for censoring by introducing an appropriate noise model and allowing a different kernel for each dimension. We evaluate this new approach for two typical qPCR datasets (of mouse embryonic stem cells and blood stem/progenitor cells, respectively) by performing linear and non-linear probabilistic PCA. Taking the censoring into account results in a 2D representation of the data, which better reflects its known structure: in both datasets, our new approach results in a better separation of known cell types and is able to reveal subpopulations in one dataset that could not be resolved using standard PCA.

**Availability and implementation:** The implementation was based on the existing Gaussian process latent variable model toolbox (https://github.com/SheffieldML/GPmat); extensions for noise models and kernels accounting for censoring are available at http://icb.helmholtz-muenchen.de/censgplvm.

**Contact:** fbuettner.phys@gmail.com

**Supplementary information:** Supplementary data are available at *Bioinformatics* online.

## 1 INTRODUCTION

### 1.1 High-throughput single-cell qPCR

To gain fundamental insights into complex cellular processes, it is necessary to observe individual cells. One such process is the transcriptional control of cell fate decisions, where it is crucial to quantify the gene expression of individual cells because cell fate decisions are made on a single-cell level. In contrast to single-cell measurements, conventional experimental techniques measure gene expression from pools of cells masking heterogeneities within cell populations, which may be important for understanding underlying biological processes (Dalerba *et al.*, 2011; Dominguez *et al.*, 2013; Guo *et al.*, 2010; Moignard *et al.*, 2013; Pina *et al.*, 2012). Recent technical advances facilitate the simultaneous measurement of tens to thousands of genes in hundreds of individual cells (Taniguchi *et al.*, 2009). As experimental techniques advance and new types of data are generated, it is important to develop sound computational methods that are able to adequately deal with uncertainties inherent in the experimental technique and to allow for a comprehensive analysis of these new types of data. Currently, the messenger RNA content of single cells can be analysed using high-throughput quantitative real-time polymerase chain reaction (qPCR) platforms, such as the Fluidigm BioMark HD, or using deep sequencing [RNA sequencing (RNA-Seq)].

**Fig. 1.**
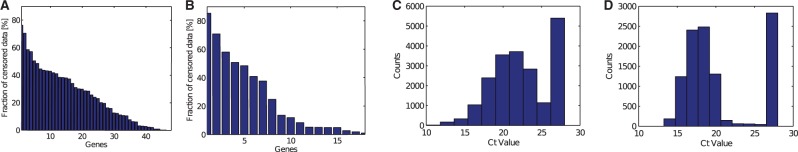
Fraction of censored data for two typical datasets: (**A**) fraction of non-detects in mESC data resolved by genes and (**B**) fractions of non-detects in blood stem cell data. Genes sorted in descending order of fraction of censored values. (**C**) distribution of Ct values for mESC data and (**D**) blood stem/progenitor cell data. The long tail of high Ct values continues until the LOD

In single-cell qPCR, RNA is extracted from single cells and complementary DNA is synthesized. This is followed by a pre-amplification step and qPCR detection. In practice, this procedure results in a limit of detection (LOD), below which gene activity cannot be quantified. Gene expression is typically measured in cycles (Ct), and depending on the analysed cell types and genes, the LOD Ct value can be defined as a 99% detection probability of the qPCR reaction and typically corresponds to ∼2–10 messenger RNA molecules per reaction chamber (Fluidigm Corporation, 2012). This corresponds to a censoring in the sense that for Ct values greater than LOD Ct, the true Ct number cannot be established. This censoring typically occurs for a large number of cells (see Fig. 1 for values of two typical datasets) and is one of the main challenges when dealing with data from single-cell qPCR experiments. For cases in which non-detection corresponds to a lack of transcription, the true Ct value would be infinity (McDavid *et al.*, 2013), whereas for cases in which non-detection corresponds to a non-negligible amount of transcription, the true underlying Ct value would be closer to LOD Ct; because the distribution of Ct values extends continuously until the LOD (Fig. 1C and D), this suggests that both scenarios can be encountered in practice.

Because high-throughput single-cell qPCR is a relatively new technique, this issue of censoring has not been addressed systematically, and simple work-arounds such as substituting all censored data points with the LOD Ct value are commonly used (Dalerba *et al.*, 2011; Guo *et al.*, 2010; Pina *et al.*, 2012).

Recently, McDavid *et al.* (2013) have systematically addressed these issues by proposing a customized approach for univariate testing of differential gene expression of single-cell qPCR data that explicitly takes the component of non-detected qPCR reaction into account. Although the authors did not address implications of the LOD for multivariate analyses such as principal component analysis (PCA), this highlights the need for new algorithms addressing statistical and analytical challenges of single-cell qPCR data.

Other sources of uncertainty on a cell-wise level such as effects due to variations in cell size can be corrected for by measuring a set of housekeeping genes and subtracting the mean expression from the measured Ct number. Similarly, uncertainties can be corrected that occur due to the batch-wise processing of cells on arrays and variations in PCR efficiency between batches.

### 1.2 PCA of censored data

A common part of multivariate analysis of single-cell qPCR data is PCA. This allows for a visualization of the variation in gene expression within and across different cell populations as well as the identification of subpopulations in a large group of heterogeneous cells (Dalerba *et al.*, 2011; Guo *et al.*, 2010). Recently, we have shown that it is desirable to also apply non-linear generalizations of PCA because this can allow for a better identification of novel subpopulations (Buettner and Theis, 2012). For many statistical methods such as regression, algorithms to deal with censored data have been established. For example, censored values can be substituted, Tobit regression can be performed or data can be deleted, treated as missing or imputed according to some probability distribution (Ballenberger *et al.*, 2012). However, it is not clear how to deal with censored data in the context of PCA, especially when there is a high fraction of censored data points. In this case, deletion can result in the loss of an unacceptably high proportion of the data. Similarly, treating the censored data as missing (Theis *et al.*, 2011) discards potentially valuable information. Substitution can yield strongly biased results, and multiple imputation results in several datasets that are difficult to combine in a single PCA (Lubin *et al.*, 2004; Uh *et al.*, 2008). Furthermore, for high fractions of censored data (as in single-cell qPCR), it is not clear how to derive adequate probability distributions (Ballenberger *et al.*, 2012; Lubin *et al.*, 2004).

**Fig. 2.**
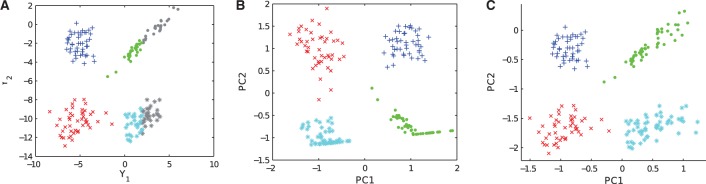
The 2D toy example (mixture of four Gaussians). In (**A**) the true values of *Y* are shown; *Y*_1_ is right censored for values >2 (shown in grey). In (**B**) a PCA is performed with all censored values substituted with two resulting in a biased representation of the data. In (**C**) a PCA taking censoring into account using an appropriate noise model is shown resulting in a more realistic representation of the data. The uncertainty inherent in the generative model is visualized using greyscale as described in Section 2.2. This uncertainty is greatest on the far right where censoring occurs

When performing PCA of censored data from single-cell qPCR, the standard approach is to substitute the Ct values of all censored data points with the same Ct value (usually LOD Ct) and perform standard PCA. In Figure 2, a toy example is used to illustrate the issues of this substitution approach. A different approach is to treat the data as censored when performing the PCA; however, to date no algorithm allowing for linear and non-linear PCA of censored data has been presented. In the following, we propose an extension of generalized PCA [Gaussian process latent variable models (GPLVMs)] allowing for censored data. When optimizing the generative mapping (including the positions of the data points in a low-dimensional latent space), we use statistically sound methods to account for the censoring such that uncertainties in the high-dimensional space are reflected in the low-dimensional visualization of the data.

We evaluate our new strategy on dealing with single-cell qPCR data on two typical datasets.

Thus, our contribution in this work is 2-fold. First, we propose a strategy for performing PCA, and probabilistic kernel PCA in general, of censored data. This allows the visualization of censored data within the commonly used framework of PCA without introducing bias due to censoring and can be used for data from a wide range of sources. Second, we present a framework on how to account for uncertainties when performing PCA of single-cell qPCR data. We quantify potential new biological insights that can be gained by accounting for censoring: in the case of single-cell qPCR data, our approach can result in PCA representations that better reflect the underlying structure of the data and allow for a better identification of biologically meaningful subpopulations.

## 2 METHODS

To derive an algorithm for PCA of censored data, we first review probabilistic dual PCA before we show how we can use this as a mathematical framework to deal with censored data.

### 2.1 Dual PCA for censored data

#### Standard PCA with Gaussian noise

Let the gene expressions in the data space be denoted by 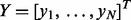, 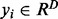, and latent variables in the low-dimensional latent space be denoted by 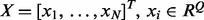, with *D* being the dimension of the data space, *Q* being the dimension of the latent space (usually 2 or 3) and *N* being the number of samples in the dataset. Then, probabilistic PCA can be written as:
(1)
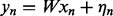

with independent and identically distributed (i.i.d.) Gaussian observation noise 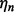: 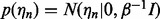 (Bishop, 2006). Although for probabilistic PCA we would marginalize over *X* and optimize the transformation matrix *W*, for dual PCA (and more generally, GPLVM), we marginalize over *W* and optimize the latent variables *X*. If we place a prior over *W* in the form of 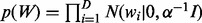 where *w_i_* is the *i-*th row of *W* and integrate over *W*, we find (Lawrence, 2004):
(2)
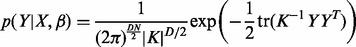

with 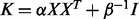. This marginalized likelihood is the product of *D* Gaussian processes with linear covariance matrix *K*. It can be shown by deriving the corresponding log-likelihood *L* with respect to the latent variables *X*; the solution is equivalent to the one obtained by solving the standard PCA problem (Lawrence, 2005). In this dual interpretation of PCA, the cell-to-cell correlation is captured by the covariance matrix *K*. If the linear kernel in *K* is substituted with a different non-linear kernel, a non-linear generalization of probabilistic dual PCA (GPLVM) is obtained. By constructing the covariance matrix using such non-linear kernel, the relationship between cells can be arbitrarily complex. We chose the commonly used radial basis function (RBF) kernel, which can be written as:
(3)
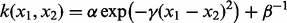

with hyperparameters α and γ.

#### Dual PCA with alternative noise models

So far the model assumes Gaussian noise 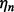 in every dimension, which is a good approach when there are neither missing nor censored data. However, if we want to perform a (dual) PCA (or GPLVM) of censored or missing data, it is necessary to use a different noise model. This can be done by introducing an additional latent variable 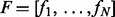 between *X* and *Y* (Lawrence, 2005):
(4)
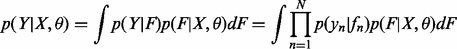



The Gaussian observation noise model used for non-censored data can then be interpreted as:
(5)
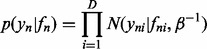



Lawrence (2005) suggested that other noise models in the form of
(6)
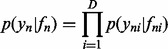

can be used. However, in the case of non-Gaussian noise models, the Gaussian approximations of 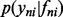 need to be found to yield a Gaussian distribution of the posterior of *F* and thus maintaining the tractability of the marginalized likelihood.
(7)
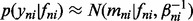



Thus, to perform PCA/GPLVM with missing and censored data, we first need to define an appropriate noise model. Next, once the (non-Gaussian) noise model is defined, we need to find a Gaussian approximation.

Using this framework to deal with missing data is straightforward: as Lawrence (2005) shows, the precision 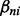 corresponding to missing values *ni* is set to 0: 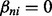.

When dealing with censored data, we need to define a more complex noise model. Here we propose to define a noise model based on the probit function (cumulative distribution function of the normal distribution)

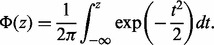



For data points *n* that are right censored at the value *b* in dimension *i*, a noise model reflecting this censoring can be defined as:
(8)
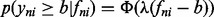

where λ controls the slope of the curve. For data that are left censored at *b_l_* in dimension *i*, we can choose the noise model accordingly [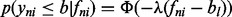]. Similarly, data that are interval censored between *b*_1_ and *b*_2__,_ can be accounted for with a noise model in the form of
(9)
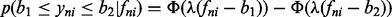



**Fig. 3.**
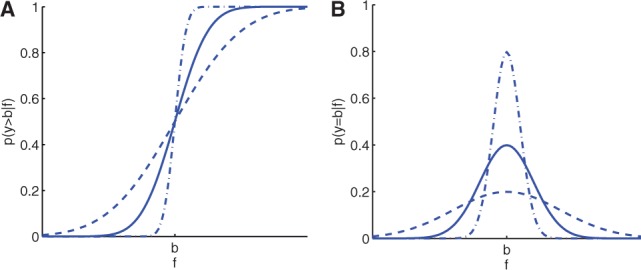
Probit noise model for three different vales of λ (**A**) and Gaussian noise model for three different values of 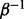 (**B**)

In Figure 3, the probit noise model for 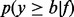 and the Gaussian noise model for 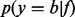 are shown. In the probit noise model, the slope/steepness of the curve is controlled by the parameter λ; similarly, the width of the Gaussian noise model is controlled by 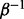.

Gaussian approximations as needed in Equation (7) can be found by using assumed density filtering (Lawrence *et al.*, 2005; Minka, 2001). Here, approximations are updated sequentially by incorporating one datum at a time. This yields an approximation *q*(*F*) to the true posterior 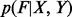 in the form of
(10)
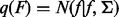

with a block-diagonal covariance matrix Σ that is built of *D* blocks 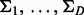. The parameters of the approximation can be calculated as:
(11)
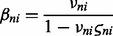

(12)
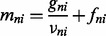

with 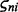 being the *n-*th diagonal element of 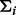, 
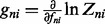
 and 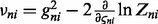. The partition function *Z_ni_* is defined as:
(13)
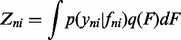



It can be shown (Lawrence *et al.*, 2005) that for the case of the probit noise model, the partition function can be calculated as:

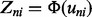

where

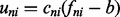

(14)
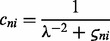



In practice, if the slope of the noise model is not fixed, we learn it together with the kernel parameters: therefore, we consider the slope of the noise model to be steep and add a white noise term to the kernel *K* in the form of 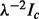 with *I_c_* being a diagonal matrix such that only the entries corresponding to censored data points are set to 1 and all other entries are set to 0—this will then result in an increase of 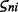 by λ and, as can be seen from Equation (14), in an equivalent description of the noise model. Note that care has to be taken because censored inputs are independent for each dimension. This means that we have to use a different kernel for each dimension because *I_c_* will be different for each dimension. However, as the marginal likelihood factorizes into *d* Gaussian processes, this extension of standard GPLVMs is straightforward, and the possibility was described earlier (Grochow *et al.*, 2004). More specifically, we choose the white noise term for dimension *d* such that 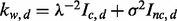 with 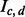 and 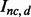 being the diagonal matrices where only those entries are set to 1 where a data point is censored and not censored, respectively. All other terms in the kernel (i.e. RBF term or linear term) were shared across all dimensions.

In summary, the generation of the PCA mapping taking censoring into account involves two major steps. First, a Gaussian approximation [Equation (7)] to the probit noise model [Equation (8)] has to be found via assumed density filtering [Equations (11) and (12)]. This yields an approximation to the log-likelihood of the model [Equation (4)]. In the second step, this approximation is maximized with respect to the latent positions *X* and the kernel parameters (including λ). For this optimization step, non-linear optimizers such as scaled conjugate gradient (Nabney, 2001) can be used.

### 2.2 Visualizing uncertainties in latent space

Performing dual PCA with the probit noise model as outlined above, yields an explicit mapping from latent space to the original high-dimensional space [Equation (4)]. When generating this mapping, not only the positions of the points in the latent space but also the parameters of the (noise) model are chosen such that censoring is accounted for. That is, the uncertainty of the data is reflected in the mapping.

Consequently, we can use this model to calculate for any point 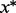 in the latent space a posterior mean 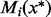 and a posterior variance 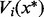 for each dimension *i* (Supplementary Note S1) (Rasmussen and Williams, 2006). For standard GPLVMs with one kernel shared across all dimensions, 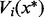 will be the same for all dimensions. In this case, it is straightforward to visualize the uncertainty of the mapping in the latent space by varying the intensity of the background pixels (background of the 2D map) (Lawrence, 2006). In our case, the posterior variance will vary across dimensions. To visualize the uncertainty across all dimensions, we use the fact that Equation (4) is a product of *D* Gaussian processes. Consequently, we can quantify the uncertainty of the mapping by calculating the product of the posterior variance across all dimensions. For visualizing this uncertainty, we then vary the intensity of the background pixels with
(15)
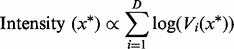



The higher the uncertainty, the darker the pixels. Black pixels correspond to the highest uncertainty.

### 2.3 PCA of censored single-cell qPCR data

Censoring in single-cell qPCR techniques occurs due to a detection limit of the qPCR reaction. This LOD both depends on the manufacturer of the machine and is experiment-specific where it can vary between different genes. Most researchers do not establish this gene-dependent LOD but use a global LOD reflecting the overall sensitivity of the qPCR machine (Guo *et al.*, 2010; Pina *et al.*, 2012). However, more objective methods to establish an LOD, such that a qPCR reaction will be found with a probability of at least 99% if the Ct value is below the LOD, can be used, too. For example, necessary experiments to do so are outlined in the manual of the popular Biomarks system (Fluidigm Corporation, 2012).

In the following, we will evaluate our new strategy on how to deal with the LOD (once it is established) when performing PCA. Therefore, we first use the standard approach where all values greater than the LOD are substituted with a particular value 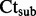. The choice of 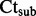 depends largely on the biological interpretation of non-detects (Supplementary Note S2). If most non-detects correspond to a genuine lack of transcription, a large value should be chosen for 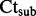 because the true underlying Ct value would be 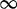 (for practical reasons, 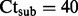 could be chosen, as a maximum of 40 Ct can typically be measured); otherwise, a value of 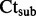 closer to LOD (or LOD) should be chosen. We followed the latter approach (setting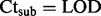), which is commonly adopted in the literature (Guo *et al.*, 2010; Pina *et al.*, 2012). Furthermore, we also explored higher values of 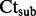 corresponding to the interpretation of non-detects as lack of transcription (Supplementary Figs. S1 and S2). Because in the substitution approach systematic uncertainties in the data in the form of censoring are ignored, in this case, a standard PCA can be performed. In addition to standard PCA, results for independent component analysis (ICA) and t-Distributed Stochastic Neighbour Embedding (t-SNE) (Amir *et al.*, 2013; van der Maaten and Hinton, 2008) using the substitution approach are shown in Supplementary Figures S1 and S2. We then compare this substitution approach to our new algorithm where PCA with the probit noise model is performed. In contrast to the substitution approach, there is no need to choose 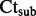 because the probit noise model accounts for uncertainties in the underlying true Ct value for non-detects. Because the non-detects are modelled separately by introducing a discrete part in the GPLVM, this can either be interpreted as a noise model for censored data or as a discrete model for genes that are ‘off’. For the probit noise model, we compare censored PCA of fixed steepness parameter λ with censored PCA where λ is optimized in the form of a parameter of a white noise kernel, as described above.

We tested the two approaches with both a linear kernel (resulting in standard PCA) and an RBF kernel to capture non-linearities in the data.

Because theoretically a maximum of 40 Ct can be measured, we used this as the upper limit in the noise model for interval censoring [Equation (9)]. This prevents the optimizer from being stuck in a local minimum where some censored data points are mapped to high Ct numbers. (In practice, this only occurred for the linear kernel with fixed λ in the blood dataset.) In each run, we followed Guo *et al.* (2010) and Moignard *et al.* (2013) and performed a cell-wise normalization by subtracting the average Ct number of the housekeeping genes from the Ct value of the gene of interest. Consequently, when data points were censored at a value *b* before normalization, this threshold was normalized accordingly for each cell (Ballenberger *et al.*, 2012).

We evaluated the different approaches on two recently published datasets. The first dataset was published by Guo *et al.* (2010). Briefly, the authors analysed the development of the mouse zygote to the bastocyst by measuring gene expression on a single-cell level. Therefore, the authors quantified the expression levels of 48 genes for 442 cells at different stages of the cellular development (1-cell stage to 64-cell stage). Cells at the 32-cell stage had undergone differentiation to either trophoectoderm (TE) cells or inner cell mass (ICM). Cells at the 64-cell stage were TE cells, primitive endoderm (PE) cells or epiblast (EPI) cells. Lables for cells at the 32- and 64-cell stages were derived from Figure 1 by Guo *et al.* (2010) by assigning each cell to the closest cluster (TE, PE, EPI, ICM). Cells from the 1-cell stage were systematically different from all other cells because of the differences in experimental conditions (Guo *et al.*, 2010). That is why we excluded all nine cells from the 1-cell stage from our analysis. More details on the dataset can be found in recent publications by Buettner and Theis (2012) and Guo *et al.* (2010).

The second dataset consists of 597 blood stem and progenitor cells, in which the expression of 24 genes was measured, including 18 transcription factors, five housekeeping genes and a cell surface marker (Moignard *et al.*, 2013). Approximately 120 individual primary cells were isolated for each population from mouse bone marrow by fluorescence-activated cell sorting (FACS). As for the ESC data, the sorted populations comprise a cellular hierarchy that gives rise to all of the mature cell types of the blood system. The haematopoietic stem cells sit atop the hierarchy and give rise to the megakaryocyte–erythroid lineage through the PreMegE progenitor or to the lymphoid-primed multipotent progenitor (LMPP). The LMPP in turn gives rise to the myeloid lineage and the lymphoid lineage through the granulocyte–monocyte progenitor and the common lymphoid progenitor (CLP), respectively (Orkin and Zon, 2008). Each population has been isolated on the basis of cell surface markers and characterized functionally either *in vivo* or *in vitro*.

In the primary analysis of both datasets an LOD of Ct = 28 was assumed.

For both datasets, we evaluated our new approach to deal with censoring for both a linear and an RBF kernel. First, we assess the effect of the probit noise model compared with the Gaussian noise model. Therefore, we make use of the generative models and calculate the posterior mean of 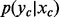 for all censored data points *c*. Furthermore, we quantified the performance of the different approaches in terms of their ability to reflect the known structure of the data by calculating the nearest neighbour error: for each cell, we established the label of its nearest neighbour in the respective 2D space; if the label differed from the original cell, we increased the nearest neighbour error count by one. We chose this metric because it is easily interpretable and commonly used in the machine learning community to quantify the performance of dimensionality reduction/visualization methods; however, as its power is limited (e.g. it does not account for newly discovered subpopulations), visual inspection as an additional performance measure is crucial.

## 3 RESULTS

In Figure 1, the fraction of censored data points in both datasets is illustrated for the different genes. It can be seen that in both datasets a considerable fraction of data is censored across some dimensions (genes), whereas for other dimensions no censoring occurred (i.e. expression of the respective gene could be detected with a Ct number below the LOD for all cells).

In the following section, we will first evaluate different approaches of PCA with censored data for the mouse embryonic stem cells (mESC) data. Next, we will repeat the evaluation with a different dataset on blood stem/progenitor cells.

### 3.1 Evaluation of censored PCA for mESC data

**Fig. 4.**
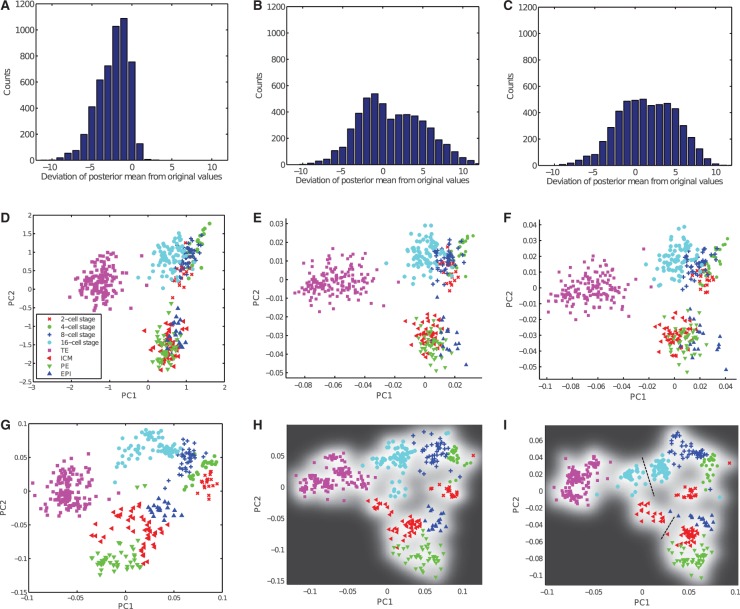
(**A–C**) Distribution of residuals between posterior means and the normalized LODs for different approaches. (**D–F**) PCA with censored data from mESC dataset. Standard PCA with substitution approach (D), taking censoring into account with probit noise model and fixed λ (E) and probit noise model with λ learnt from data (F). (**G–J**) GPLVM with RBF kernel for mESC data. Standard GPLVM with substitution approach (G), taking censoring into account with probit noise model and fixed λ (H) and probit noise model with λ learnt from data (I). In (I) the dashed lines indicate two distinct subpopulations at the 16-cell stage and ICM

The result of a standard PCA where all censored values are substituted with LOD (as described in Section 2.3) is shown in Figure 4D. This method was used in the original publication and yielded a nearest neighbour error of 124. In the original high-dimensional data space, the nearest neighbour error was 10. Note that this error was calculated using the substitution approach.

Although TE cells can be clearly distinguished from all other cell types, early cells from 2- to 8-cell stages are strongly overlapping. Similarly, there is a strong overlap between ICM cells and PE/EPI cells.

Next, we compare the substitution method with our new algorithm for censored PCA. In Figure 4E and F, the results for a fixed 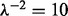 are shown together with the representation where λ was optimized together with the other kernel parameters.

For a quantitative analysis of the effects of the different noise models, we used the generative mapping from the latent space to the high-dimensional space to calculate the posterior means given all censored data points in the low-dimensional space. We then calculated the residuals between the posterior means and the respective normalized LOD. In Figure 4A–C, it can be seen that when using the substitution approach, the censored values are mapped consistently to values lower than the normalized LOD. In contrast, when our new approach is used, a large fraction of censored data points is mapped to values greater than the normalized LOD, which is in better agreement with the ground truth. When λ was learnt from the data by optimizing it in the form of a kernel parameter, the maximum a posteriori estimate was 15.3 for 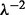. Compared with a fixed value for 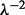 of 10, censored data points were mapped closer to the normalized LOD. It can be seen that taking into account the censoring results in an improved mapping where EPI cells can be separated better from ICM/PE cells than in the standard method. This is reflected in lower nearest neighbour errors of 113 and 88 for fixed λ and learnt λ, respectively.

We also evaluated our new approach for an RBF kernel, which allows non-linearities to be taken into account. The resulting mappings are shown in Figure 4G–I.

It can be seen that in the non-linear case, the separation between different time points and cell times is comparable between the substitution approach and our new approach. This is also reflected in the similar nearest neighbour errors of 11, 12 and 10, respectively.

**Fig. 5.**
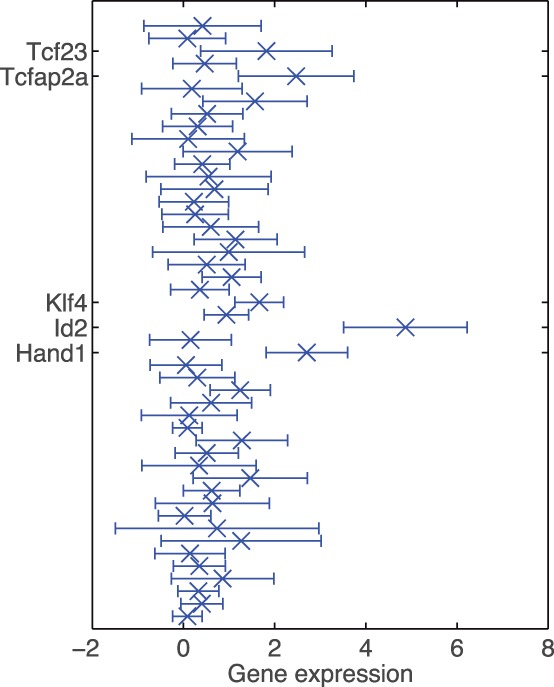
Difference in gene expression between the two subclusters at the 16-cell stage for different mappings. The error bars show the variation of gene expression within the smaller subcluster (one standard deviation in each direction). For convenience, genes with the greatest differences are labelled in the plots

However, it can be seen that the ICM cells as well as cells from the 16-cell stage are separated into two clusters when the censoring is accounted for. This leaves room for interpretation. When comparing mean gene expression for the two subclusters in the 16-cell stage, we found that expression in Id2 and Klf4 differed considerably between the two subclusters (*P*-values after Wilcoxon rank sum test 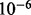 and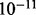, respectively, Fig. 5). This is in good agreement with previously reported experimental results from Guo *et al.* (2010), who show that Id2 is the earliest markers for outer cells. Similarly, when comparing mean gene expressions in the two subclusters of ICM cells, we found that they differed significantly in expression of Fgf4 (*P* = 0.01, Wilcoxon rank sum test). This is also in good agreement with previously reported results showing differential expression of Fgf4 in the early ICM (Guo *et al.*, 2010). Thus, when allowing for non-linearities and taking censoring into account, it was possible to correctly represent the structure of the data for all cell types and resolve subpopulations that could not be revealed when not accounting for censoring. In Supplementary Figure S3, the nearest neighbour errors for all approaches to perform a PCA of the mESC data set are shown. In Supplementary Figure S1, we show the results for the substitution approach with other multivariate methods for different choices of 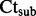. All approaches yielded higher nearest neighbour errors than the GPLVM with probit noise model.

### 3.2 Evaluation of censored PCA for blood stem/progenitor cell data

To evaluate the potential benefits of our new approach for PCA of censored data with a second independent biological dataset, we next applied our new analysis tools to a recently generated single-cell gene expression dataset for five fluorescence-activated cell sorting-sorted populations of blood stem and progenitor cells.

**Fig. 6.**
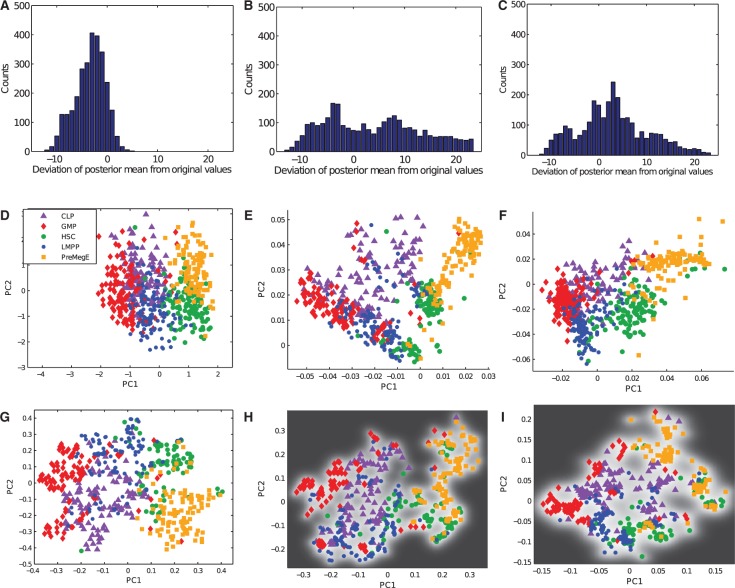
(**A–C**) Distribution of residuals between posterior means and the normalized LODs for different approaches. (**D–F**) PCA with censored data from blood dataset. Standard PCA with substitution approach (D), taking censoring into account with probit noise model and fixed λ (E) and probit noise model with λ learnt from data (F). (**G–J**) GPLVM with RBF kernel for blood data. Standard GPLVM with substitution approach (G), taking censoring into account with probit noise model and fixed λ (H) and probit noise model with λ learnt from data (I). The background intensity indicates the relative uncertainty of the mapping with black pixels corresponding to the highest uncertainty of the mapping

As for the mESC dataset, we first compared standard PCA with the substitution approach to censored PCA with the probit noise model. Results are shown in Figure 6D–F. It can be seen that by accounting for censoring in the data, a better separation is achieved between most cell types; this occurs most clearly for CLPs and granulocyte–monocyte progenitors. Consequently, nearest neighbour errors decreased from 254 errors with the standard substitution approach to 193 and 217 when censoring was accounted for by fixing λ and learning λ, respectively. As for the mESC dataset, we found that the censored PCA approach yielded better posterior mean values for censored data points than the standard approach using substitution (Fig. 6A–C).

We also used an RBF kernel to evaluate the non-linear PCA with censored data. Results for the different approaches are shown in Figure 6G–I. When accounting for the censoring, the nearest neighbour error was reduced and a better separation between CLPs and LMPPs than for the substitution approach was possible. Nearest neighbour error rates for all approaches are summarized in Supplementary Figure S3. In Supplementary Figure S2, we show results for the substitution approach with other multivariate methods for different choices of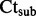. All approaches yielded higher nearest neighbour errors than the GPLVM with probit noise model.

## 4 DISCUSSION

Conventional approaches for PCA of censored data where values beyond the detection limit are substituted with the detection limit can yield strongly biased results. We have proposed a novel approach for performing dual PCA of censored data. Our new approach resulted in a mapping between low-dimensional and high-dimensional space such that more censored data points were mapped correctly to values greater than the detection limit. It was previously shown that for single-cell qPCR data, it is crucial to explicitly model the population of non-detects when performing a statistical test of univariate differential expression (McDavid *et al.*, 2013). To date no approaches for dealing with this issue for multivariate analyses such as PCA have been proposed. We evaluated our new approach for two different real-world datasets comprising measurements of single-cell qPCR data. For both datasets, the PCA representations better reflected the known structure of the data when the censoring was explicitly considered. We evaluated using a linear as well as a non-linear kernel, and for both datasets accounting for non-linearities resulted in better visualizations. In contrast to using a linear kernel (i.e. PCA), this comes at the price of losing interpretability—although in the linear case loadings can be easily visualized in a bi-plot, in the non-linear case this is more difficult because loadings change across the 2D plot. Whether trading off interpretability for complexity is beneficial depends highly on the dataset under consideration and any non-linearities present. In the context of single-cell qPCR data, our analyses suggest that a non-linear kernel is necessary to capture the typical complex dependency structure of such data.

For linear kernels (corresponding to standard PCA) as well as for non-linear kernels (allowing for interactions), our new approach yielded considerably lower nearest neighbour error rates with reductions of up to 29% in the linear case. Furthermore, in the case of mESC data, the structure of subpopulations was reflected better in the case when censoring was taken into account in the non-linear case: in contrast to non-linear probabilistic PCA with the substitution approach, two subpopulations corresponding to cells from the 16-cell stage with high Id2 expression and cells in the ICM with high Fgf4 expression could be identified. These known subpopulations were previously identified in a univariate analysis of cells from the same cell stage. However, this standard approach has several drawbacks because it can become unfeasible when too many genes are measured simultaneously. Furthermore, only univariate patterns can be identified, whereas important information may lie in multivariate patterns, which could be defined by the differential expression of several genes. Finally, when analysing univariate distributions or correlations between two genes for cells from the same cell stage, the identified subpopulations cannot be put in context with other cells from other cell stages. In contrast, when performing a probabilistic (kernel) PCA of all cell stages, it is possible to identify complex multivariate subpopulations, and by simultaneously displaying all cells, the PCA plot provides an intuitive illustration of the relation between all cell populations.

This was achieved by implementing a GPLVM with different kernels for each dimension. Censoring was accounted for by a probit noise level. The steepness parameter of the probit function was learnt together with other kernel parameters, resulting in a parameter-free approach for PCA of censored data.

Although our approach was designed for accounting for uncertainties in single-cell qPCR data, related issues can be found in single-cell RNA-Seq data. In contrast to single-cell qPCR, however, high levels of technical noise are present in all commonly used protocols for single-cell RNA-Seq (Brennecke *et al.*, 2013). This technical noise is particularly strong for low levels of expression and dominates all other uncertainties (like censoring). Although these uncertainties are inherently different from the censoring found in single-cell qPCR, the flexible framework of Gaussian processes allows us to account for these uncertainties in a straightforward manner by using an additional term in the (Gaussian) noise model reflecting the technical noise, which can be estimated using the approach suggested by Brennecke *et al.* (2013). Although single-cell RNA-Seq data are generated in the form of read counts, it is crucial to perform normalization steps accounting for different cell sizes, different sequencing depth and, depending on the protocol, different transcript lengths (Brennecke *et al.*, 2013; Yan *et al.*, 2013). Such normalization can be performed by calculating reads per kilo base per million (RPKM) and fragments per kilobase of exon per million fragments mapped (FPKM) or using DEseq-inspired normalization procedures (Anders and Huber, 2010; Brennecke *et al.*, 2013). After normalization, gene expression is measured on a continuous scale such that—after an appropriate variance-stabilizing transformation (e.g. log-transformation)—GPLVM can be applied without modification. Because efficient implementations allow fast processing of datasets with tens of thousands of genes and hundreds of cells without overfitting, it is a promising tool for analysing such datasets.

The main drawback of our proposed approach is that it scales cubically with the number of cells, which may be prohibitive when the number of analysed cells is very large (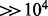). Although standard GPLVMs are time-consuming, too, significant speed-ups can be achieved because of sharing the kernel across all dimensions and using a spherical noise model. However, if necessary, approximations resulting in sparse covariance matrices commonly used in Gaussian process literature could be applied for our framework, too. For the application to single-cell qPCR data, we found that this was not necessary because computation times were in the order of only a few hours on a standard laptop. We acknowledge that this is a considerable increase of time compared with standard PCA, which can be performed when using the substitution approach to deal with censored data. In applications with only a small fraction of censored data points, this rather large increase in runtime may result in only minor changes of the PCA representation, and simpler approaches such as the substitution approach or treating the data as missing may be a valid alternative if runtime is an issue. However, in the case of single-cell qPCR data, we have shown that taking censoring into account avoids a potential bias in low-dimensional representations due to the censoring. This in turn can result in better biological insights: first, our approach can yield a better separation of different cell types; second, it may even reveal new biologically meaningful subpopulations that may be obscured because of a bias introduced by the censoring. When designing single-cell qPCR experiments, the quantification of heterogeneities and the reliable identification of new subpopulations are often key goals. That is why we believe that our approach will be of interest for many practitioners working with censored data, especially in the field of high-throughput single-cell qPCR.

## 5 CONCLUSION

We have presented a new approach for performing probabilistic PCA for censored data within the framework of GPLVMs. Therefore, we implemented an appropriate noise model and allowed different kernels for each dimension. We showed that for single-cell qPCR data with a high fraction of censored data points, the resulting probabilistic (kernel) PCA representations reflected the true structure of the data better than conventional approaches. In two real-world datasets, known cell types could be better separated when censoring was taken into account, and in one dataset several distinct subpopulations could be revealed that could not be resolved with standard PCA.

*Funding*: F.B. and F.J.T. acknowledge funding from the European Research Council (starting grant ‘LatentCauses’). V.M. and B.G. acknowledge funding from Leukaemia and Lymphoma Research (LLR), Cancer Research UK (CRUK) and the Biotechnology and Biological Sciences Research Council (BBSRC). V.M. is funded by a Medical Research Council studentship.

*Conflict of Interest*: none declared.

## Supplementary Material

Supplementary Data
