# Supplementary material for: Probabilistic PCA of censored data: accounting for uncertainties in the visualization of high-throughput single-cell qPCR data
Source: Bioinformatics. 2014 Mar 10;30(13):1867–75. doi: 10.1093/bioinformatics/btu134 (PMC4071202; doi:10.1093/bioinformatics/btu134)
Supplement: Supplementary Data [file supp_30_13_1867__index.html]

Probabilistic PCA of censored data: accounting for uncertainties in the visualisation of high-throughput single-cell qPCR data — Probabilistic PCA of censored data: accounting for uncertainties in the visualization of high-throughput single-cell qPCR data — Probabilistic PCA of censored data: accounting for uncertainties in the visualization of high-throughput single-cell qPCR data — Supplementary Data 

# Probabilistic PCA of censored data: accounting for uncertainties in the visualization of high-throughput single-cell qPCR data

## Supplementary Data

files

**Files in this Data Supplement:**

- Supplementary Data - pdf file
