# Supplementary material for: Probabilistic PCA of censored data: accounting for uncertainties in the visualization of high-throughput single-cell qPCR data
Source: Bioinformatics. 2014 Mar 10;30(13):1867–75. doi: 10.1093/bioinformatics/btu134 (PMC4071202; doi:10.1093/bioinformatics/btu134)
Supplement: Supplementary Data [file supp_btu134_supplementAdobe.pdf]

Supplementary Material for: Probabilistic PCA of censored data: accounting for uncertainties in the visualisation of high-throughput single-cell qPCR data

Supplementary note 1: Visualising uncertainties in latent space

Posterior mean and covariance of a Gaussian Process can be computed as follows:

$$M_i(x^*) = k_*^T K_i^{-1} y_i \tag{1}$$
$$\Sigma_i^* = k_{**} - k_*^T K_i^{-1} k_* \tag{2}$$

with i indicating the ith dimension, k_* being the $N \times 1$ vector of covariances $(\sigma(x_1, x^*), \dots \sigma(x_N, x^*))$ and $k_{**} = \sigma(x^*, x^*)$ which can be calculated using the previously learnt kernel hyperparameters [4]. In the linear case (standard PCA) $k_{**} = \alpha x^* x^{*T} + \beta^{-1}$ so that the uncertainty can be dominated by the first term in eq. 2 and will increase the further x^* is away from the origin. However, in the non-linear case, when $k_{**} = \alpha \exp\left(-\gamma(x^* - x^*)^2\right) + \beta^{-1} = \alpha + \beta^{-1}$ the posterior variance will be depend only on the second term, such that it is smallest when x^* is close to data-points. Hence, as the uncertainty is most meaningful and interpretable in the non-linear case, we only visualise it in the non-linear case.

Supplementary note 2: Biological interpretation of non-detects

While it is well-known that in many single-cell qPCR experiments a large number of non-detects occur, their biological interpretation remains unclear. Recently, McDavid et al. [2] have suggested that non-detection reflects a lack of transcription. They base this suggestion on the observation that in their dataset they did not observe any Ct values close to the limit of detection. In contrast, in the data-sets analysed in this study, we do find instances of this (Fig 1 C and D), suggesting some of the non-detects correspond to a non-negligible expression. While most researcher substitute non-detects with LOD Ct for multivariate analyses [3, 1], we now explore the possibility of non-detects corresponding to zero expression and perform PCA, ICA and t-SNE for using higher Ct values in the substitution approach.

Supplementary Figures

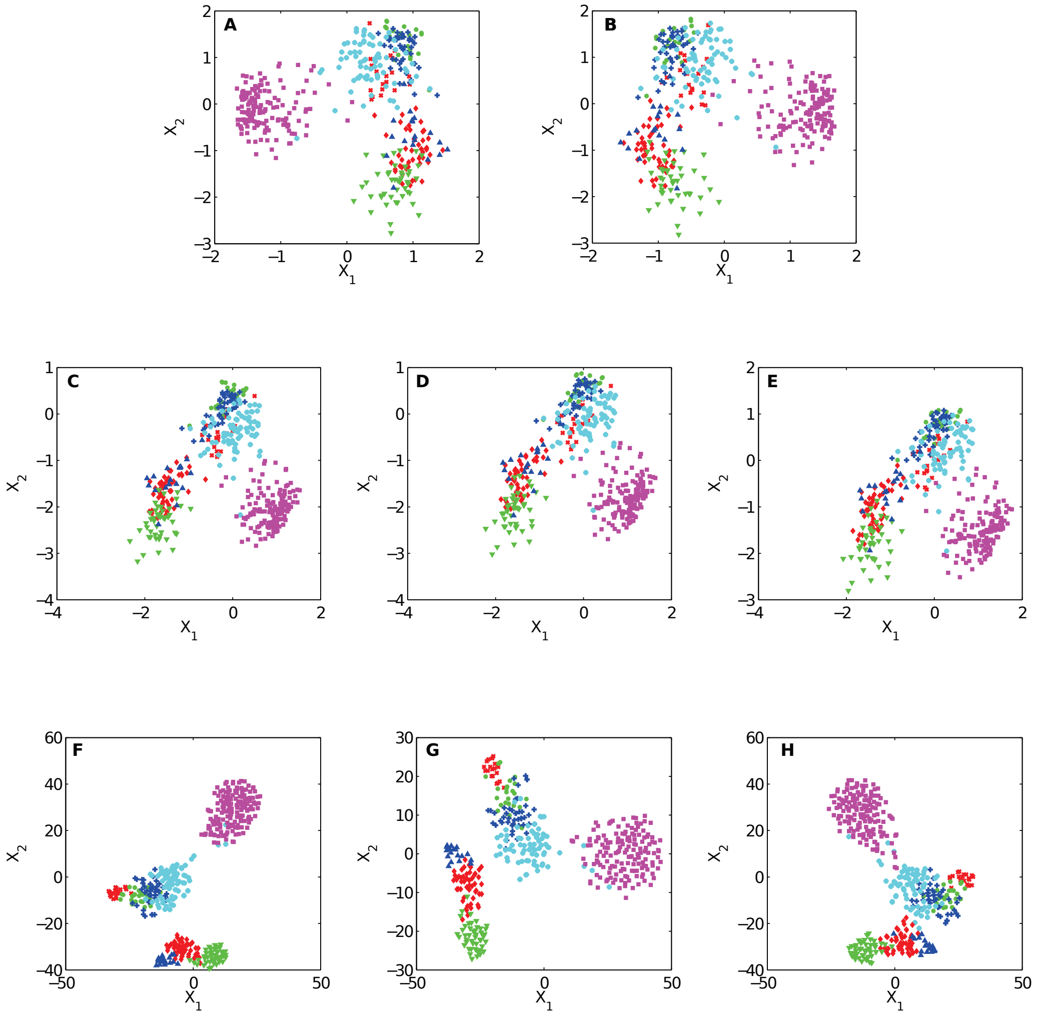

Supplementary Figure 1: Susbtitution approach for mESC data. A, PCA with Ct_{sub} = 40 (NNE=121), B, PCA with Ct_{sub} = 100 (NNE=122), C, ICA with Ct_{sub} = 28 (NNE=112), D, ICA with Ct_{sub} = 40 (NNE=121), E, ICA with Ct_{sub} = 100 (NNE=122), F, t-SNE with Ct_{sub} = 28 (NNE=48), G, t-SNE with Ct_{sub} = 40 (NNE=52), H, t-SNE with Ct_{sub} = 100 (NNE=57)

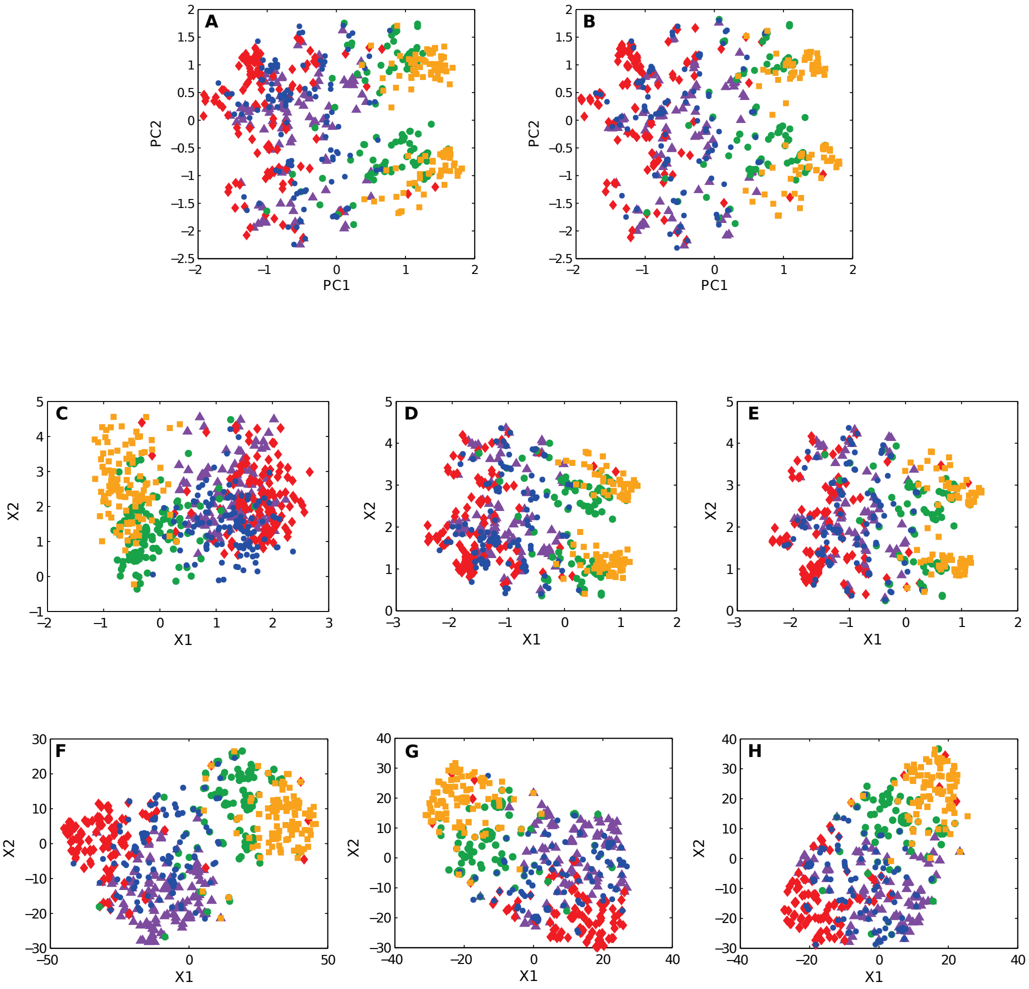

Supplementary Figure 2: Susbtitution approach for blood stem/progenitor cell data. A, PCA with $Ct_{sub} = 40$ (NNE=241), B, PCA with $Ct_{sub} = 100$ (NNE=231), C, ICA with $Ct_{sub} = 28$ (NNE=260), D, ICA with $Ct_{sub} = 40$ (NNE=241), E, ICA with $Ct_{sub} = 100$ (NNE=228), F, t-SNE with $Ct_{sub} = 28$ (NNE=170), G, t-SNE with $Ct_{sub} = 40$ (NNE=175), H, t-SNE with $Ct_{sub} = 100$ (NNE=177)

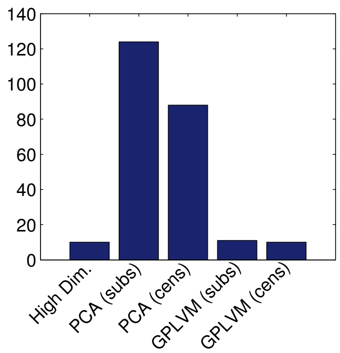

(a) mESC data

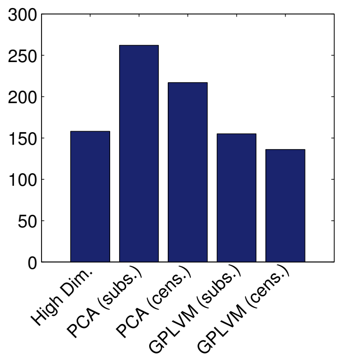

(b) Blood stem/progenitor cell data

Supplementary Figure 3: Summury of nearest neighbour errors for the approaches presented in the main text. (a) mESC data and (b) blood stem/progenitor cell data.

References

[1] Guoji Guo, Mikael Huss, Guo Qing Tong, Chaoyang Wang, Li Li Sun, Neil D. Clarke, and Paul Robson. Resolution of cell fate decisions revealed by single-cell gene expression analysis from zygote to blastocyst. *Dev Cell*, 18(4):675–685, Apr 2010.

[2] Andrew McDavid, Greg Finak, Pratip K. Chattopadyay, Maria Dominguez, Laurie Lamoreaux, Steven S. Ma, Mario Roederer, and Raphael Gottardo. Data exploration, quality control and testing in single-cell qpcr-based gene expression experiments. *Bioinformatics*, 29(4):461–467, Feb 2013.

[3] Cristina Pina, Cristina Fugazza, Alex J. Tipping, John Brown, Shamit Soneji, Jose Teles, Carsten Peterson, and Tariq Enver. Inferring rules of lineage commitment in haematopoiesis. *Nat Cell Biol*, 14(3):287–294, Mar 2012.

[4] C.E. Rasmussen and C.K.I. Williams. *Gaussian processes for machine learning*. Adaptive computation and machine learning. MIT Press, 2006.
